# Recent developments in microbial production of high-purity galacto-oligosaccharides

**DOI:** 10.1007/s11274-022-03279-4

**Published:** 2022-04-20

**Authors:** Anna Maráz, Zoltán Kovács, Eric Benjamins, Melinda Pázmándi

**Affiliations:** 1 Department of Food Microbiology, Hygiene and Safety, Institute of Food Science and Technology, Hungarian University of Agriculture and Life Sciences, H-1118, Somlói út 14-16, Budapest, Hungary; 2 Department of Food Process Engineering, Institute of Food Science and Technology, Hungarian University of Agriculture and Life Sciences, Budapest, Hungary; 3 Ausnutria Dairy Ltd, Zwolle, Overijssel Netherlands

**Keywords:** Galacto-oligosaccharides (GOS), Microbial purification, Fermentation strategies, Prebiotics

## Abstract

Galacto-oligosaccharides (GOS) are used as prebiotic ingredients in various food and pharmaceutical formulations. Currently, production of GOS involves the enzymatic conversion of lactose by transgalactosylation using β-galactosidase. The purity of the resulting product is low, typically limited to up to 55% GOS on total carbohydrate basis due to the presence of non-reacted lactose, and the formation of by-products glucose and galactose. In industrial practice high-purity GOS is manufactured by removing the unwanted mono- and disaccharides from raw GOS with simulated moving bed (SMB) chromatography. This purification step is associated with high processing cost that increases the price of pure GOS and limits its marketability. The last decades have witnessed a growing interest in developing competitive biotechnological processes that could replace chromatography. This paper presents a comprehensive review on the recent advancements of microbial GOS purification, a process commonly referred to as *selective fermentation* or *selective metabolism.* Purification strategies include: (i) removal of glucose alone or together with galactose by lactose negative yeast species, that typically results in purity values below 60% due to remaining lactose; (ii) removal of both mono- and disaccharides by combining the fast monosaccharide metabolizing capacity of some yeast species with efficient lactose consumption by certain lactose positive microbes, reaching GOS purity in the range of 60–95%; and (iii) the application of selected strains of *Kluyveromyces* species with high lactose metabolizing activity to achieve high-purity GOS that is practically free from lactose and monosaccharides.

**Graphical abstract:**

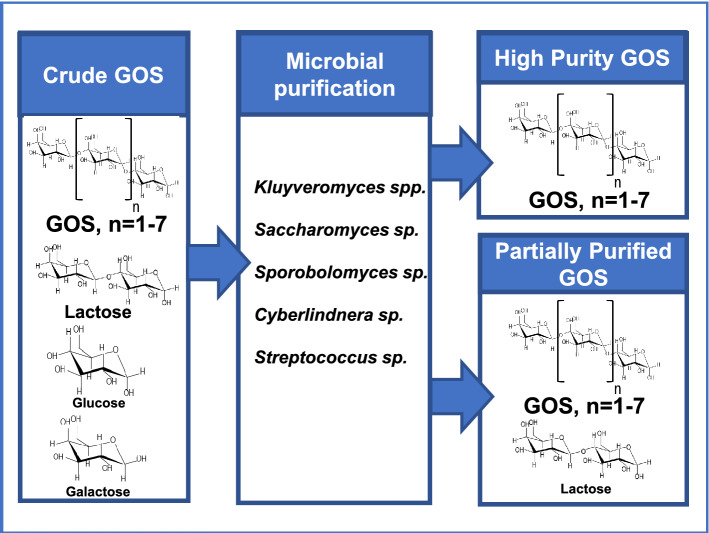

## Introduction

GOS are short-chain, non-digestible carbohydrates with functional properties offering a variety of health benefits (Sijbers et al. 2020). Their prebiotic activity has been demonstrated by several studies, using both in vitro and in vivo approaches (Hong et al. 2016). Alongside their favorable physiological effects, they have excellent technological properties, such as high solubility, low viscosity, good pH and temperature stability, and pleasant texture with a sweet flavor (Torres et al. 2010).

GOS typically consist of a glucose molecule at the reducing terminus and 1–7 galactose units connected by various types of glycosidic linkages, such as β(1,2), β(1,3) β(1,4) and β(1,6) (de Almeida and Maitan-Alfenas 2021). Although the chemical structure of GOS shows very little similarity to that of oligosaccharides present in breast milk, GOS are known to resemble some prebiotic effects of human milk oligosaccharides (Salli et al. 2019). In fact, infant nutrition represents the largest market for GOS. They are also applied as prebiotic components in various functional foods including dairy products and their analogs, snacks, nutrition bars and beverages. Also, GOS are increasingly used in feedstock (Rentas et al. 2020), pharmaceuticals, dietary supplements (Ghosh et al. 2020; Ambrogi et al. 2021b) and cosmetics (Hong et al. 2017).

The GOS market has witnessed a significant growth since the release of the first commercial GOS product by Yakult Pharmaceuticals Industry Co. Ltd from Japan in 1989. The global GOS market size is estimated to be 570 million USD in 2021 and expected to grow with a compound annual growth rate of about 6% from 2021 to 2026 (Market Data Forecast Inc. 2021).

GOS occur naturally and can be extracted from biological sources (Kim et al. 2003). Their isolation however is not cost effective due to their relatively low concentration. Although production of GOS compounds is possible by acidic hydrolysis of lactose at elevated temperatures, the chemical synthesis is not preferred due to the possible formation of unwanted compounds and the lack of product specificity (Ambrogi et al. 2021a). At present, biosynthesis for industrial GOS production is exclusively used from lactose, which is generated in large quantities in the dairy industry.

## Galacto-oligosaccharides

GOS are produced by the enzymatic conversion of lactose using β-galactosidases of various origins such as bacteria, yeasts and filamentous fungi. Several bioprocesses employing whole cells and (semi-)purified enzymes in free or immobilized form have been successfully implemented for GOS production (Illanes et al. 2016). The source of enzyme used in the reaction greatly affects the type of the linkages and the chain lengths of the resulting GOS (Schultz et al. 2021). The conversion is a kinetically controlled reaction accompanied by competition between the unwanted hydrolysis and the desirable transgalactosylation. To avoid hydrolysis, the reaction is performed at high substrate concentrations (typically above 300 g/L lactose) (Pázmándi et al. 2018).

Commercial GOS products are usually manufactured in batch fashion, starting with the preparation of concentrated lactose solution at high temperature (typically above 50 °C) to overcome solubility limitations of lactose. Then, transglycosylation is initiated in a stirred tank reactor with the addition of β-galactosidase at optimal reaction conditions (pH, temperature and substrate concentration). After GOS synthesis, the enzyme is inactivated by heating and/or acidic treatment. Further processing steps may include decolorization with active carbon, demineralization with ion-exchange chromatography, and concentration by evaporation to obtain crude GOS in syrup form (Scott et al. 2016). Powders are usually formulated by dosing some carrier materials such as maltodextrin or proteins into crude GOS during the spray-drying process to overcome the low glass transition temperature caused by the large amounts of monosaccharides.

The biocatalytic process results in crude GOS that is a mixture of saccharides with relatively low GOS content. In addition to the oligosaccharides, it contains considerable amounts of non-reacted lactose and undesired monomers (glucose and galactose) as side products of the transgalactosylation reaction. Under optimized settings the obtained yield rarely exceeds 55% GOS on total carbohydrate basis (Vera et al. 2016; Scott et al. 2016). A notable drawback of the biosynthesis is that the remaining lactose and the generated monosaccharides carry no prebiotic function but a significant caloric value.

## Prebiotic function of GOS fractions

Prebiotic effect of GOS fractions with a degree of polymerization (DP) ≥ 3 has been extensively demonstrated (Bouhnik et al. 1997; Tuohy et al. 2005; Roberfroid et al. 2010; Maathuis et al. 2012). Crude GOS products contain disaccharides in different quantities depending on the type of enzyme used. Coulier et al. (2009) identified eight non-lactose disaccharides in the DP2 fraction of a commercial GOS product (Vivinal GOS, FrieslandCampina Domo B. V., Beilen, The Netherlands), which comprised 27.4 weight percent (wt%) of the crude GOS in addition to 22.5 wt% monosaccharides, 10 wt % lactose and 40.1 wt % DP3-DP8. Four of the non-lactose DP2 compounds were structural isomers of lactose, such as the functional isomer allolactose. Lactulose (β-D-Gal-(1,4)-D-Fru), which has a proven prebiotic effect (Gibson et al. 2004; Tuohy et al. 2005; Roberfroid et al. 2010), was also present in as low as 7 wt % of the non-lactose DP2 fraction. Daily dosage of lactulose in the range of prebiotics (5 g) for 5 days exerted the full beneficial prebiotic effect for Bifidobacteria, Lactobacilli and Anaerostipes in a computer-controlled in vitro model of the human large intestine (Bothe et al. 2017). The prebiotic potential of other non-lactose disaccharides was also studied by several in vitro fermentation experiments using faecal sludge. Sanz et al. (2005) demonstrated that 4β-galactobiose and 6β-galactobiose possess higher prebiotic index than lactulose, while Rodriguez-Colinas et al. (2013) found that the mixture of allolactose and 6β-galactobiose supported the growth of Bifidobacteria but not lactic acid bacteria (LAB). The complex prebiotic nature of these disaccharides has not been proven yet by in vivo digestibility tests.

Van Leeuwen et al. (2016) compared the composition of Vivinal GOS with six other commercial GOS products and identified five non-lactose disaccharides comprising galactose-galactose or glucose-galactose dimers. A common component in all GOS products was a β-D-Gal-(1,x)-D-Glc dimer with different molar ratios across the various products. These findings indicate that non-lactose disaccharides are substantial and high ratio components of commercial GOS products. However, it could be rarely predicted which of these compounds possess prebiotic characteristics and should be kept as prebiotic components, or which do not possess such traits and should be removed during purification. It would be advisable to analyse the DP2 components and select which fulfil all three accepted criteria for classification as a prebiotic: (1) resistance to gastric acidity, hydrolysis by mammalian digestive enzymes and GI absorption; (2) fermentation by intestinal microbiota; (3) selective stimulation of the growth and/or activity(ies) of one or a limited number of intestinal bacteria beneficially associated with health and well-being (Gibson et al. 2004). Without comprehensive characterization only lactulose could be considered as a prebiotic DP2 GOS component presently.

## High-purity GOS

There is an emerging commercial interest for GOS products that contain decreased level of or free from digestible carbohydrates by the human intestine. Several types of purified GOS products can be distinguished based on their carbohydrate composition. Obviously, products free from digestible carbohydrates can be obtained by the removal of both lactose and the monosaccharides from crude GOS. Commercial products with a content of 90 w/w% GOS or higher are considered as high-purity GOS (Scott et al., 2016). As the incidence of lactose intolerance increases, there is a growing demand for lactose-free GOS products. However, the presence of lactose is accepted or even preferred in certain applications. For instance, partially purified GOS ingredients for infant formulas are typically manufactured by removing only the monosaccharides from crude GOS (Pázmándi et al. 2020; Vera et al. 2022). Industrially, purification of crude GOS is done by SMB chromatography (Mueller et al. 2021). To overcome the high processing cost associated with chromatography, several attempts have been made at developing competitive separation techniques. These include adsorption on activated carbon followed by regeneration with ethanolic mixtures (Boon et al. 2000; Hernández et al. 2009), conversion of glucose into gluconic acid by glucose oxidase (Cheng et al. 2006; Todea et al. 2021), selective oxidation of residual lactose into lactobionic acid by cellobiose dehydrogenase (Splechtna et al. 2001; Maischberger et al. 2008), fractionation by nano- and diafiltration (Pruksasri et al. 2015; Córdova et al. 2017), ethanolic precipitation (Sen et al. 2011) and supercritical extraction (Montañés et al. 2010). Despite some promising results, at this point none of the investigated techniques have been proved to be robustly effective in replacing chromatography at industrial scale. In recent years selective metabolism of non-GOS carbohydrates by various microorganisms has received special attention as one of the most promising technologies to decrease downstream operational costs (Kruschitz and Nidetzky 2020). Detailed analysis of these downstream techniques including selective fermentation, as well as summary of their advantages and drawbacks have been reviewed currently by Vera et al. (2022).

## Advanced metabolic characteristics of microorganisms aimed at GOS purification

Application of microorganisms for the selective metabolism of non-GOS sugar components of crude GOS would require overcoming the following barriers: (i) reduction in the mass ratio of cells to sugars, (ii) no or limited addition of nutrients required for cell cultivation, (iii) dilution of crude GOS products prior to fermentation in a rate that allows economic concentration after purification and (iv) minimization of the generation of unwanted metabolites, which would require increased efforts and costs for downstream processing.

Development of industrially reliable processes for selective removal of lactose, glucose and galactose from crude GOS by microorganisms should consider several issues. Among them metabolic characteristics, stress tolerance against crude GOS and fermentation environments, food safety aspects and economic viability are the most important. Microbial cultures with documented use in food production have divergent metabolic routes including efficient utilisation of non-GOS saccharides, as well as tolerance and adaptation to the eventually harsh food extrinsic and intrinsic environmental factors. Bourdichon et al. (2012) published an updated inventory of safe microbial cultures used in or isolated from food fermentations, listing as much as 195 bacterial species and 69 species of yeasts and moulds. Species harbouring probiotic strains could also be the sources of safe microorganisms to be used for GOS purification.

Concomitant removal of lactose, glucose and galactose from crude GOS is a great challenge for microorganisms, lactose utilization being the bottleneck of this process. Lactose catabolism is highly influenced by the regulation of β-galactosidases, as their activity is strongly inhibited by galactose and—in a much lesser extent—glucose (Park and Oh 2010; Eberhardt et al. 2021).

## Metabolism of non-GOS sugars from crude GOS by LAB

In the inventory of microbial food cultures published by Bourdichon et al. (2012), the most efficient lactose utilizing bacterial species belong to the genera of *Bifidobacterium, Lactobacillus, Pediococcus* and *Lactococcus.* The most abundant genus is the *Lactobacillus* with 83 species. Lactose fermentation is a complex process, while galactose catabolism is a variable characteristic among the LAB species, even within a unique species. Iskandar et al. (2019) gave an excellent overview of lactose and galactose utilization of LAB, including the internalization, subsequent metabolism of these carbohydrates and the genetic basics of the connected biochemical and physiological processes. In addition, a comparative genomic analysis of the lactose and galactose utilization-specific Leloir and Tagatose-6-P pathways has been performed in around 200 strains. They concluded that majority of the investigated *Lactobacillus* strains carry the Leloir pathway (gal) genes alone or in combination with the Tagatose-6P pathway (lac) genes, while not any pathway-specific genes could be detected in a small portion of the strains. At the same time, *Lactococcus* strains harboured only pathway-specific genes. Leloir pathway genes were always accompanied with that of the Tagatose-6-P pathway, while the Tagatose-6P pathway genes occurred alone. They found that the copy number of certain genes was also variable among or within the species. The high rate of genomic variability of the genes responsible for the galactose and lactose metabolism seems to be responsible for the wide metabolic diversity of LAB from this respect. Genomic annotation of lactose and galactose catabolism genes of certain LAB strains as potential candidates of crude GOS purification would open the possibility for the selection and genetic improvement of more robust strains.

GOS fermentation is connected to the type and activity of ß-galactosidases, although the lactose fermenting ability for efficient GOS hydrolysis is strongly influenced by internalization of GOS with different DPs (Gänzle and Follador 2012). Pázmándi et al. (2021) selected several lactose fermenting *Lactobacillus* strains, which did not utilize GOS molecules > DP2 in in vitro fermentation experiments. It is to be noted that besides the dairy product starters, probiotic strains also occurred among the selected strains, indicating that GOS utilization ability of potential probiotic strains should be analyzed more carefully, preferably by in vivo digestive tests.

## Metabolism of non-GOS sugars from crude GOS by yeasts

“Food grade” lactose utilizing yeasts represent the most promising pool of fungi to be assessed for GOS purification. The number of potentially safe lactose utilizing yeast species was estimated to be as high as 143 by Pázmándi et al. (2020). When the lactose utilization capacity of the selected strains was determined, it was found that the strains could function optimally with 20–30 g/L lactose, while 50 g/L already decreased the growth rates. Among the lactose positive yeasts, *Kluyveromyces* species are most efficient in lactose assimilation. The Crabree-negative *K. lactis* and *K. marxianus* utilize lactose not only by aerobic respiration but also ethanolic fermentation, while the other two lactose utilizing species of the genus, *K. nonfermentans* and *K. wickerhamii* assimilate but do not ferment lactose. Pázmándi et al. (2020) found that *K. lactis* and *K. marxianus* metabolized the lactose and glucose content of crude GOS simultaneously, although depletion of glucose was always faster than that of the lactose. Galactose utilization was concomitant with that of the lactose.

## Stress tolerance of GOS purifying microorganisms

During metabolism of non-GOS sugars, several environmental stress factors target the GOS purifying microorganisms. Most significant stress factors are the hyperosmotic pressure—as the consequence of economically feasible concentration of crude GOS -, the oxidative stress and the inhibitory effect of certain fermentation end products like organic acids and ethanol. De Angelis and Gobbetti (2004) surveyed the physiological and molecular mechanisms of environmental stress responses in dairy and probiotic *Lactobacillus* spp. Based on the molecular mechanisms and proteomics of stress responses they were able to elaborate several strategies for the generation of improved, more robust strains with enhanced stress tolerance. Since the time of this publication considerable progress in the genomics, proteomics, bioinformatics and molecular techniques took place (Sun et al. 2015), which increased the reliability of and opened up new possibilities for the more rational use and precise genetic modification of LAB for biotechnological purposes.

Stress tolerance of yeasts is outstanding among the microorganisms, especially those causing spoilage of foods, including dairy products. Some spoilage species (e.g. *K. marxianus, K. lactis* and *Debaryomyces hansenii*) are, however, beneficial in the production of certain fermented foods (Maráz and Kovács 2014). Lane et al. (2011) demonstrated that *K. marxianus* strains have high but different extent of resistance to high osmotic conditions and moderate cell wall integrity stresses with some strains showing resistance for multiple stress factors. Therefore, extended strain selection and genetic improvement could be a good strategy for possessing more robust lactose utilizing yeast strains for GOS purification purposes.

## Overview of the proposed GOS purification procedures by microorganisms

**Fig. 1:**
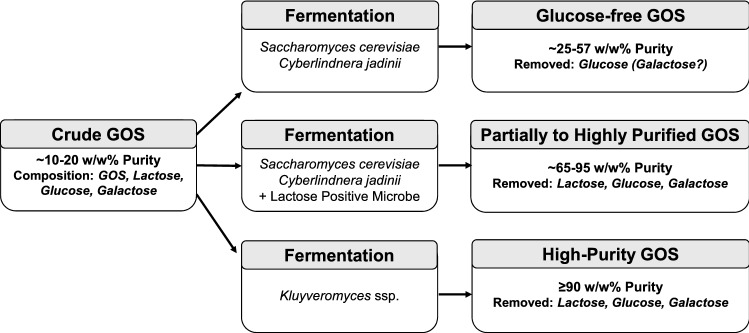
Current advances of GOS purification via selective metabolism of non-GOS sugars by key microorganisms. End products of purification represent the main types of commercial GOS

Fermentation-based GOS purification methods can be categorized into three types, depending on the removed saccharide fractions and the applied microorganisms: (A) glucose and occasionally galactose removal by lactose negative species (e.g. *Saccharomyces cerevisiae* and *Cyberlindnera jadinii*); (B) Removal of mono- and disaccharides from GOS with the aid of *S. cerevisiae* or *C. jadinii* and a lactose positive microbe. This approach combines the fast monosaccharide-removal capacity of *S. cerevisiae* and *C. jadinii* with the efficient lactose metabolism of the other strain; (C) Application of selected strains of *Kluyveromyces* species with high lactose metabolizing activity to achieve high-purity (mono- and disaccharide-free) GOS. An outline of the crude GOS purification strategies is illustrated in Fig. 1. Table 1 shows an overview of the relevant publications categorized in the above mentioned groups.

**Table 1:** Types of crude GOS purification with various microbes, characteristics of the applied fermentation processes and final purity achieved at the end of fermentation

Category^a^	Microorganism^b^	Crude GOS (w/w%)	Fermentation conditions^c^	Type of sugar depletion^d^	Nutrient supplement^e^	Fermentation time (hours)	GOS purity^f^ (%)	References
A/	*S. cerevisiae*	45	Batch	B	NA	32	57	Goulas et al. (2007)
	*S. cerevisiae*	20	Batch, IC	B	NA	4	37	Li et al. (2008)
	*S. cerevisiae*	16.7	Batch	B	NA	10	49	Hernández et al. (2009)
	*S. cerevisiae*	–	SSP	B	NA	24	40	Aburto et al. (2016)
	*S. cerevisiae*	–	SSP	B	NA	8	25.7	Aburto et al. (2018)
	*C. jadinii*	15	Batch	C	5 g/L YE	24	49	Pázmándi et al. (2020)
B/	*S. cerevisiae* + *Sc. thermophilus*	10	Batch	B	NA	70	> 95	Giacomelli et al. (2015)
	*S. cerevisiae* + *Sm. singularis*	–	SSP	B	NA	60	> 85	Saravanan et al. (2017)
	*S. cerevisiae* + *K. lactis*	20	Batch	B	NA	32	65	Srivastava and Mishra (2019)
	*C. jadinii* + *K. lactis*	10	Batch	C	5 g/L YE	72	92	Pázmándi et al. (2020)
C/	*K. marxianus*	20	Batch	C	5 g/LYE	30	98	Cheng et al. (2006)
	*K. lactis*	20	Batch, IC	B	NA	18	97	Li et al. (2008)
	*K. marxianus*	20	Batch	B	NA	48	100	Guerrero et al. (2014)
	*K. lactis*	10	Batch	B	NA	15	> 95	Sun et al. (2016)
	*K. marxianus*	30	Batch, IC	C	3 g/L YE + inorg N, S, P	26	100	Tokošová et al. (2016)
	*K. lactis*	20	Batch	B	NA	19	96	Santibáñez et al. (2016)
	*K. lactics*	10	Batch	C	5 g/L YE	72	92	Pázmándi et al. (2020)
	*K. marxiaunus*	10	Batch	C	5 g/L YE	72	100	Pázmándi et al. (2020)
	*K. lactis*	10	Batch	B	NA	8	75	Zheng et al. (2021)

^a^A/: fermentation with lactose negative microbes B/: combination of lactose negative and positive microbes C/: fermentation with lactose positive microbes

^b^Abbreviation of the genus names: *K.: Kluyveromyces, S.: Saccharomyces, Sm.: Sporobolomyces, Sc.: Streptococcus, C.: Cyberlindnera*

^c^IC: immobilized cells; SSP: Simultaneous synthesis and purification

^d^B: Bioconversion, C: Catabolism

^e^YE: Yeast extract; NA: Not applicable

^f^GOS purity: ratio of the ΣDP3-DP6 fractions and total amount of carbohydrates at the end of the fermentation

In the first group, the goal is the selective removal of glucose and preferably galactose. Generally, the result of fermentation largely depends on the metabolic activity of the applied strain. Hernández et al. (2009) were able to remove only glucose, while in the experiments of Goulas et al. (2007) both glucose and galactose were entirely used up. Efficient glucose depletion was achieved by a selected *C. jadinii* strain by Pázmándi et al. (2020).

To improve the efficiency of the traditional batch fermentation, Li et al. (2008) proposed a process with immobilized *S. cerevisiae* cells, by which monosaccharide-free GOS was achieved. Aburto et al. (2016) introduced a simultaneous synthesis and purification (SSP) method, combining free *Aspergillus oryzae* ß-galactosidase with *S. cerevisiae* cells, and produced GOS free from both glucose and galactose. This group later attempted co-immobilization of the enzyme and *S. cerevisiae* cells (Aburto et al. 2018). Although the product contained residual glucose and galactose, the process was advantageous in terms of reusability and the applied cell load in comparison to the batch setup.

The second group of GOS purification attempts comprised selective fermentations by a consortium of microbes. Giacomelli et al. (2015) proposed a three-step process, using *S. cerevisiae*, *Streptococcus thermophilus* and *S. cerevisiae* in a sequential fashion to remove glucose, lactose and galactose from crude GOS. Although high-purity GOS was achieved, the process involved multiple pH control and cell removal stages, making it labor-intensive. A two-step approach was introduced by Srivastava and Mishra (2019), who applied *S. cerevisiae* and *K. lactis* cells in a sequential fashion for the removal of mono- and disaccharides. Although the purity of the product was low, as none of the non-GOS compounds were removed entirely, both S*. cerevisiae* and *K. lactis* cells could have been recycled up to 10-times. Pázmándi et al. (2020) proposed a two-step process to produce high-purity GOS, starting with glucose removal by *C. jadinii*, followed by lactose and galactose removal by *K. lactis*. An SSP setup was used by Saravanan et al. (2017) for the production of GOS by *Sporobolomyces singularis* cells, and monosaccharide removal by *S. cerevisiae*. This procedure resulted in monosaccharide-free GOS with a low amount of residual lactose.

Up to this point, the most successful attempts—in terms of GOS purity and the ease and speed of the removal process—were the application of *Kluyveromyces* strains, belonging to *K. lactis* (Li et al. 2008; Sun et al. 2016; Santibáñez et al. 2017; Pázmándi et al. 2020; Zhang et al. 2021) and *K. marxianus* (Cheng et al. 2006; Guerrero et al. 2014; Tokošová et al. 2016; Pázmándi et al. 2020). In all cases, high-purity GOS was produced, free from monosaccharides and containing no or only negligible amounts of all types of disaccharides (prebiotic and non-prebiotic alike).

Although the use of alternative techniques of the traditional batch process (e.g. fed-batch fermentation, SSP, cell immobilization) plays a big role in advancing fermentation technologies in general, their application in selective GOS fermentation have remained limited to laboratory-scale. In the surveyed GOS purification processes (Table 1), the most commonly used reaction-setup was batch fermentation (Cheng et al. 2006; Guerrero et al. 2014; Sun et al. 2016; Tokošová et al. 2016; Pázmándi et al. 2020; Zhang et al. 2021). Santibáñez et al. (2017) improved the fermentation rate and the purity of the final product, when lactose content of crude GOS was hydrolyzed prior to the fermentation either by a *K. lactis* derived commercial ß-galactosidase or permeabilized *K. lactis* cells. Yeast cells were immobilized in Ca-alginate and polyvinyl alcohol (PVA) by Li et al. (2008) and Tokošová et al. (2016), respectively, to improve the re-usability of the batch process.

Pázmándi et al. (2021) proposed a new application of LAB to purify crude GOS by fermentation of a highly diluted GOS syrup with GOS non-fermenting *L. paracasei* and *L. plantarum* strains. The end-product was free from mono- and disaccharides but due to the low GOS concentration (ca. 5.3 g/L), it was not suited to be concentrated economically. The fermentation broth was nutritionally safe, considering the quality and quantity of the lactic fermentation-derived organic acids as well as the nutrients added to support the growth of cells. The lactic fermented prebiotic broth could contribute to the formulation of functional beverages.

Majority of the purification processes can be considered as biotransformation. In most cases, relatively high cell loads were used, the g_cell_/g_carbohydrate_ ratio was 0.02–0.25 for *S. cerevisiae*; 0.05–0.1 g_cell_/g_carbohydrate_ for co-fermentations; and 0.15–0.7 for *Kluyveromyces*-based fermentations across the proposed processes, although Li et al. (2008) used an outstanding 2.5 g_cell_/g_carbohydrate_ ratio.

A traditional fermentation approach with low initial cell number was used by Cheng et al. (2006), Tokošová et al. (2016) and Pázmándi et al. (2020). In these cases, it was necessary to supplement the crude GOS-based media with growth enhancing nutrients.

The main advantage of bioconversion is that nutrient-addition is not required. However, this method necessitates production of high number of cells prior to the purification process. The fermentation-based approaches do not require great cell-mass production before fermentation, but the non-consumed supplements need to be removed downstream. None of the presented works included the analysis of the economic feasibility of the procedures, which would be influenced by two main factors: the costs of the cell mass production and the necessary downstream purification steps (Kovács et al. 2013; Scott et al. 2016).

A further limiting factor of these studies is that, in most cases, the lactose and non-lactose disaccharide components of the DP2 GOS fraction were not distinguished. Only a small number of trials were able to monitor lactose and non-lactose components within the DP2 fraction simultaneously. Cheng et al. (2006) observed that during crude GOS fermentation with *K. marxianus*, both lactose and non-lactose were depleted. Santibáñez et al. (2017) and Giacomelli et al. (2015) reported that *K. lactis* and *Sm. singularis* strains, applied in their experiments, consumed lactose but did not metabolize other DP2 components. However, in most publications it was reported that all disaccharides were catabolized by the *K. lactis* and *K. marxianus* strains. Since certain non-lactose disaccharides possess prebiotic effects, future attempts should consider screening for and improving strains that are not able to metabolize the DP2 GOS compounds, aiming to increase the yield of the process in terms of prebiotic saccharide fractions.

## Abbreviations

DP: Degree of polymerization
Gal: Galactose
Glc: Glucose
GOS: Galacto-oligosaccharides
*K*: *Kluyveromyces*
LAB: Lactic acid bacteria
*S*: *Saccharomyces*
*Sm*: *Sporobolomyces*
SMB: Simulated moving bed
SSP: Simultaneous synthesis and purification
